# Small Object Detection *via* Pixel Level Balancing With Applications to Blood Cell Detection

**DOI:** 10.3389/fphys.2022.911297

**Published:** 2022-06-17

**Authors:** Bin Hu, Yang Liu, Pengzhi Chu, Minglei Tong, Qingjie Kong

**Affiliations:** ^1^ Department of Compute Science and Engineering, School of Electronic Information and Electrical Engineering, Shanghai Jiao Tong University, Shanghai, China; ^2^ Department of Dermatology, Shanghai Ninth People’s Hospital Affiliated to Shanghai Jiao Tong University School of Medicine, Shanghai, China; ^3^ Department of Laser and Aesthetic Medicine, Shanghai Ninth People’s Hospital Affiliated to Shanghai Jiao Tong University School of Medicine, Shanghai, China; ^4^ College of Electronics and Information Engineering, Shanghai University of Electric Power, Shanghai, China; ^5^ Riseye Research, Riseye Intelligent Technology (Shanghai) Co., Ltd., Shanghai, China

**Keywords:** medical image detection, object detection, small object, pixel level balance, blood cell detection

## Abstract

Object detection technology has been widely used in medical field, such as detecting the images of blood cell to count the changes and distribution for assisting the diagnosis of diseases. However, detecting small objects is one of the most challenging and important problems especially in medical scenarios. Most of the objects in medical images are very small but influential. Improving the detection performance of small objects is a very meaningful topic for medical detection. Current researches mainly focus on the extraction of small object features and data augmentation for small object samples, all of these researches focus on extracting the feature space of small objects better. However, in the training process of a detection model, objects of different sizes are mixed together, which may interfere with each other and affect the performance of small object detection. In this paper, we propose a method called pixel level balancing (PLB), which takes into account the number of pixels contained in the detection box as an impact factor to characterize the size of the inspected objects, and uses this as an impact factor. The training loss of each object of different size is adjusted by a weight dynamically, so as to improve the accuracy of small object detection. Finally, through experiments, we demonstrate that the size of objects in object detection interfere with each other. So that we can improve the accuracy of small object detection through PLB operation. This method can perform well with blood cell detection in our experiments.

## 1 Introduction

With the development of artificial intelligence technology, deep learning based on CNN (Convolutional Neural Network) has been widely used in medical image processing field. Using computer aided technology to analyze and process medical images can assist doctors doing qualitative and quantitative analysis of diseases, thereby improving the accuracy and reliability of medical diagnosis greatly. Medical image detection is one of the main tasks in the field of medical image processing. Many medical institutions in the world have rapidly entered this field. Medical image detection has been combined with artificial intelligence technology for a long time. As early as 1993, CNN has been used for lung nodule detection. In 1995, the technology was also applied to detect micro-calcification in mammography.

Medical detection technology has been continuously developed by applying CNN and other deep learning methods (McInerney and Terzopoulos, 1996; Handels et al., 2013; Litjens et al., 2017) to various medical images with different imaging mechanisms. For example, Setio et al. detected lung nodules in 3D chest CT scans and extracted 2D patches in nine different orientations centered on these candidates (Golan et al., 2016). Ross et al. utilized CNN to improve three existing CAD systems for the detection of colonic polyps, sclerosing spinal deformity, and lymphadenopathy in CT imaging (Roth et al., 2016). In recent years, object detection technology has been widely used in pathology (Janowczyk and Madabhushi, 2016), especially in blood cells detection (Yang et al., 2017; Pan et al., 2018; Fujita and Han, 2020). Detecting blood cells can assist diagnosing many kinds of diseases, such as diagnosing breast cancer by detecting mitosis or lymphocytes (Cire ş an et al., 2013; Zhang et al., 2022). Object detection technology is constantly applied into application scenarios of medical image processing, and thus bringing more commercial value.

Object detection is mainly aimed at locating and identifying objects in different positions in the image. In medical image detection, the detection of small objects gets more attentions than the detection of large objects. For example, lesions are identified by detecting whether there are tiny abnormalities in the images, determining whether a patient is likely to develop the disease. In particular, in the detection of blood cells, some types of cells are rarer and smaller than others, but they play an indispensable and key role in the diagnosis of diseases. In such tasks, the large adjacent objects in the image can be ignored, but the detection of small objects is very important. However, object detection for small objects is precisely a more difficult task. Objectively speaking, small objects contain low pheromones and human beings have the disadvantage in recognizing small objects. These reasons bring a higher challenge to the detection task.

Previous research work mainly focused on how to enhance the detection model’s extraction of the feature of small objects, and through some methods to solve the unbalance of the samples. In addition, there is also the use of rotation detection with angle factors (Yang et al., 2019a; Yang et al., 2019b; Qian et al., 2019; Yang and Yan, 2020) to better approximate the true position of small objects. However, it is rarely mentioned that under the multi-object detection task, there is a possibility of interference among the multiple objects within one image. In the objective logic of human observation, people can see large objects most intuitively among objects of various sizes, but it is easy to ignore the existence of small objects, let alone the recognition of the small objects. In the detection model, theoretically, it is also necessary to consider the mutual interference between objects of different sizes during the training process, and the contribution of objects with various sizes to the training loss are different. Therefore, it is meaningful to study the interference of large and small objects, and use some means to alleviate such interference and guide the model to the optimization direction of small object detection.

We studied the interference between objects of different sizes in the training process, and designed a weight coefficient called PLB weight to adjust the training effect of the model. The size of the detected object is characterized by the size of the detection rectangle. We use the number of pixels contained in it as the calculation input of the weight coefficient. In the process of model training, this coefficient can be used to dynamically adjust the training loss of each object with various sizes, so that the model can be training to the direction of improving the accuracy of small object detection.

Our innovations and contributions to this work are summarized as follows:

1) Instead of setting fixed empirical values before training, we dynamically set the loss weights for objects of different sizes during the training process of the detection model.
2) With our proposed method PLB, the training trend of two-stage detector can be adjusted and the detection accuracy of small objects can be further improved.
3) Our method can be combined with other methods to improve the detection effect, bringing more potential capacity for some medical applications which need higher detection effect of small objects than bigger ones, such as blood cell detection.

## 2 Related Work

Current detection models are divided into two categories: two-stage detectors and one-stage detectors. Two-stage detection network is represented by the RCNN series (Girshick et al., 2014; Girshick, 2015; He et al., 2015; Ren et al., 2015; Dai et al., 2016), the second category is represented by the YOLO series (Redmon et al., 2016; Redmon and Farhadi, 2017; Redmon and Farhadi, 2018; Bochkovskiy et al., 2020) and SSD series (Liu et al., 2016; Shrivastava et al., 2016; Fu et al., 2017; Jeong et al., 2017; Li and Zhou, 2017; Shen et al., 2017). Among them, the former adopts the RPN network (Girshick, 2015). When performing localization and recognition tasks, candidate rectangular boxes are proposed in the RPN phase. In the second stage, the candidate proposals are adjusted and the objects in the boxes are identified. One-stage detectors use an end-to-end deep neural network, and the model structure is simpler than two-stage detectors, bring a faster computing speed, so that it is more suitable for some time-sensitive application scenarios. But for the improvement of detection accuracy, one-stage detector usually weaker than two-stage detector.

No matter which detection model is used, the CNN model is used as the feature extractor to obtain the feature space of the train set. With the continuous development of CNN models in recognition tasks, especially the ResNet model (He et al., 2016) and the DenseNet model (Huang et al., 2017), it has been confirmed that the CNN model has a high accuracy and universality for feature extraction in classification tasks. In terms of improving the overall accuracy of the detection model, a deeper CNN can be used as the backbone network to extract the image features (Zhang et al., 2022). Attention mechanism such as SENet (Hu et al., 2018) is used to improve the sensitivity of the model to channel features. The model can be adjusted through EfficientNet (Tan and Le, 2019) by adjusting the depth, width and pixel accuracy in the model to optimize the overall performance, such as EfficientDet (Tan et al., 2020). In addition, other methods such as NMS (Neubeck and Van Gool, 2006) and BN (Li et al., 2019) can be used to optimize the detection model comprehensively.

The cost of manual labeling of medical datasets is more expensive, and the acquisition and labeling of datasets is more difficult than other scenarios. Therefore, for some incompletely labeled datasets, some methods are also needed to improve the accuracy of object detection. Unsupervised active learning methods can be applied into this task to improve detection performance (Changsheng et al., 2019). Such as Active Learning Matrix Sketching (ALMS) (Li et al., 2021) which is used to do simultaneous sample and feature selection in an unsupervised setting. These methods aim to improve the effectiveness of the latent feature space (Li et al., 2022), so that the detection model can achieve more stable and good performance.

Improving the detection accuracy of small objects is a more difficult challenge. For the detection improvement of small target objects, rotation detection is also an effective method. Traditional detection models generally use horizontal rectangular boxes as labels for localization tasks. However, for small objects, the rotating detection boxes with an angle can more closely approximate the real position (Yang et al., 2021a; Yang et al., 2021b; Yang and Yan, 2022). Small objects have higher sensitivity requirements to position, and rotation detection can bring better training effects for the detection of small objects (Yang et al., 2020; Yang et al., 2021c; Yang et al., 2022).

In the detection model based on deep neural network, with the deepening of the network, the image features can be extracted better to fit our detection task. But in the feature space at the end of the model, the represented receptive field is getting bigger and bigger, while the features corresponding to the small objects may disappear. FPN network (Lin et al., 2017a; Pan et al., 2018) is a good solution to this problem. In this network structure, middle layers in the feature extraction process are reserved and combined with the upper and lower layers, so that the feature of small objects will not disappear with the deepening of the network. Finally multi-layer feature vectors are obtained by FPN. Among them, the low-dimensional feature has a smaller receptive field for small objects which is biased towards the shape features of the object, while the high-dimensional feature has a larger receptive field which is biased towards the semantic features. The FPN network improves the feature extraction effect especially for small objects as it can retain more features. As a result, the FPN network can effectively improve the detection accuracy of small objects.

Different kinds of imbalances within the training samples are also the reasons for the difficulty of detecting small objects. These imbalances mainly include the imbalance of the object categories, and the imbalance of the proportion of small objects and large objects in the samples. In addition, the imbalance between the foreground and background is also an important factor that disturbs the training effect. In the detection model, the corresponding weights can be set for each category in the data set through a prior knowledge, and the loss in the training process is weighted to adjust for the category imbalance of samples. For the spatial imbalance of the detection task, some data augmentation techniques (Pan et al., 2018; Kisantal et al., 2019) can be used to deal with this problem. For example, copy the small objects and paste them at different positions in the image multiple times to increase the proportion of small objects. Besides, data augmentation of training samples can also be performed through image fusion (Li et al., 2013; Xu et al., 2013) and image adversarial generation (Fang et al., 2020) techniques. This expansion method can alleviate the imbalance of samples in space. For the imbalance between foreground and background, the weight of difficult and easy samples can be adjusted reference to the theoretical method Focal Loss (Lin et al., 2017b). The main idea of this method is to use an appropriate function to measure the contribution of hard-to-classify and easy-to-classify samples to the total loss for a better training effect.

## 3 Proposed Method

### 3.1 Overall Structure of Our Model

**FIGURE 1:**
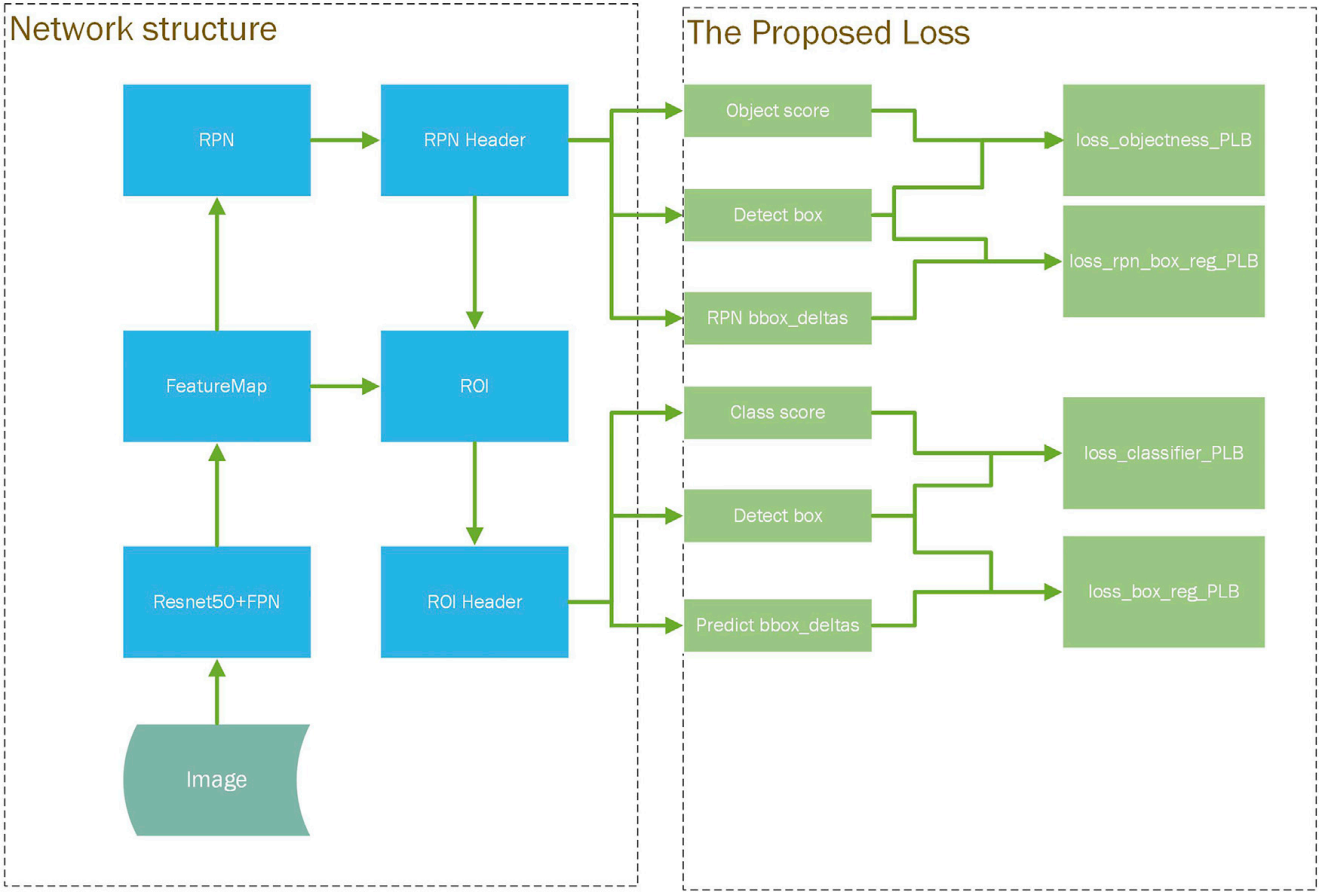
Overview of our approach design.

Figure 1 shows the structure of our detection model. In our new model, the network structure of the model is mostly like a general faster-rcnn structure. Our design is that the “RPN Header” module and the “ROI Header” module in the detection will output the coordinate of the detect box which will be put in the calculation process of the loss function. We modified the calculation of the loss function in a general two-stage detection model, using the size of the “Detect box” as a weight factor for the training loss contributed by each detect box. Through the computational design of the weight factor, we can appropriately adjust the loss contribution of each object with different sizes, and then improve the detection effect of small objects during the training process.

In the structure of our detection model, there are four loss functions that can be adjusted in this way, which correspond to the training effect of all classification task and localization task in the two-stage detection model. As the four new loss function shown in the right of Figure 1, these components use the weight factors to adjust the contribution of each detect box to the corresponding original loss.

The total loss of the detection is calculated by weighting the above four components and setting a certain weight coefficient for the original Smooth L1 Loss (Ren et al., 2015) and Cross Entropy Loss (Ren et al., 2015). For different application scenarios, the PLB (Pixel Level Balancing) operation can selectively adopt a combination strategy of these four new loss functions.

In our detection model, the training loss for each image is defined as:

$$\begin{array}{l} \;\;\;\;\;L\left(p_{i},t_{i}\right)=\frac{1}{N_{cls\_rpn}}\sum_{i}^{n}Plb_{i}L_{cls\_rpn}\left(p_{i},p_{i}^{*}\right)+\lambda \frac{1}{N_{reg\_rpn}}\sum_{i}^{n}p_{i}^{*}Plb_{i}L_{reg\_rpn}\left(t_{i},t_{i}^{*}\right)+\\ \frac{1}{\;N_{cls\_roi}}\sum_{i}^{n}Plb_{i}L_{cls\_roi}\left(p_{i},p_{i}^{*}\right)+\lambda \frac{1}{N_{reg\_roi}}\sum_{i}^{n}Plb_{i}L_{reg\_roi}\left(t_{i},t_{i}^{*}\right) \end{array}$$ (1)

where p_i_ and t_i_ are the predicted category and position results, λ is a parameter to weight the classification and the localization task. In the RPN stage, only the loss of the foreground object is calculated. The total loss function can optionally use the new loss function weighted by PLB weight factors to replace the original loss function components. If using the original loss function, just set the PLB weights to 1.

### 3.2 Design of Pixel Level Balance Factor

In the training process of the detection model, due to the different sizes of the objects, the sensitivity of the training to the size of the objects is different, and there exists a potential mutual interference. Pixel level balance refers to adjust the weight coefficients for the training loss caused by each object under inspection when multiple objects appear in the same image, and considering their different sizes as a factor to change their mutual interference. In particular, it can be assumed that large objects will adversely affect the detection of small objects, so that in the model, the detection accuracy of small objects is further reduced. On the contrary, we can actively guide the model to change towards the optimization of small object detection by adjusting the weight coefficient of each inspected object.

The number of pixels of the inspected object can be used to measure the sensitivity of its size to detection. During the training process, the size of each object is measured by the specific rectangular box area. For the selection of the rectangular box, the predicted box in the model training process can be used, or the ground-truth labeled box that best matches the candidate box can be used, they can be used as the representation of the object size.

The pixel level balance factor is defined as follows:

$$\mathrm{area\_mean}=\frac{\sum_{i}^{n}\mathrm{box\_area}}{n}$$ (2)

$$\mathrm{PLB\_weight}=\frac{\mathrm{area\_mean}*2}{\mathrm{area\_predict + area\_mean}}\;$$ (3)

where n is the number of detection boxes after filtered for the loss in this training, “area_predict”: the area of the predicted box or labeled box.

If the contribution of the loss is determined according to the number of pixels, it can be considered that an object of average size has a balance factor of 1. Taking the detection accuracy of small objects as the goal, in the above formula, when “area_predict” approaches to 0, PLB value is equal to 2, which increases the weight coefficient of small objects; when “area_predict” approaches to the largest object among the n inspected objects, assumed that sizes of the rest objects is close to 0, then

$$\mathrm{PLB\_weight}=\frac{2*\frac{\mathrm{area\_max}}{n}\;}{\frac{\mathrm{area\_max}}{n}\mathrm{+ area\_max}}=\frac{2}{\mathrm{n+1}}$$ (4)

Obviously, when there is only one object under detection, that means n is 1, then PLB weight is 1. Larger the object is, the PLB weight of the object is getting smaller.

The value scope of the PLB factor is in (2/n+1, 2), and the object with the average size has a corresponding weight of 1. It can be considered that after adding the pixel balance factor as a weight, each inspected object, regardless of its size, will contribute equally to the loss function. Using such a design, the training of the new model has a better effect compared to the original method on optimizing the detection accuracy of small objects.

### 3.3 Loss Function Combined With Pixel Level Balance

Taking “faster_resnet50_fpn” as a basic model, for its classification loss and border regression loss, PLB operations can be integrated in the four loss functions.

For the loss of classification, we still use the Cross Entropy Loss function as the loss standard of the model. But we need to calculate the pixel level balancing factor according to the size of the object corresponding to each detected box. Then we use it as the weight for multiple classification loss in a batch of images. The pixel level balancing factor can be calculated by the predicted box or its corresponding labeled box. The implementation logic of the function is shown in Algorithm 1.

Algorithm 1 Cross Entropy Loss With Pixel Level Balancing

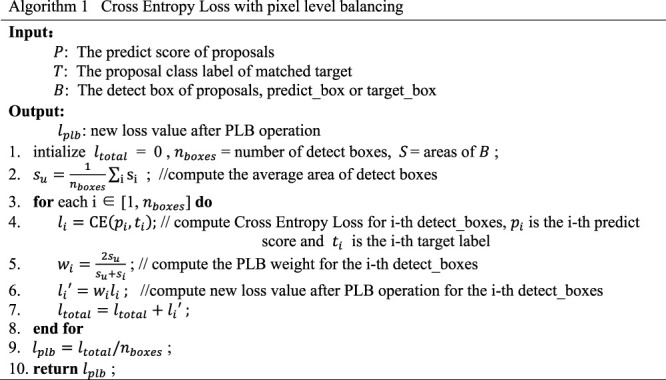

For the border regression loss function, Smooth L1 Loss is also used as the loss standard of the model, but it is necessary to calculate the pixel level balance factor according to the size of objects corresponding to each predicted box, and then calculate the pixel level balance factor of multiple objects of different sizes within a batch of images. Then we use it as the weight for localization loss for each object in our new function. The specific implementation process is shown in Algorithm 2.

Algorithm 2 Smooth L1 Loss With Pixel Level Balancing

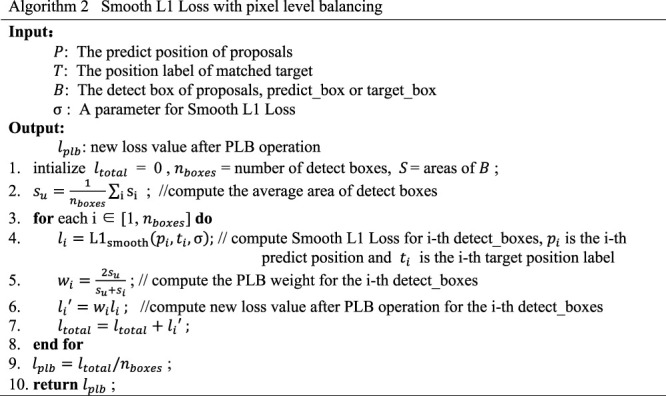

It can be seen that in the new loss function, the prediction information of the box is also added to the calculation of the classification loss, so that the classification loss and the width and height of the detected box have a certain correlation. The border regression loss will focus on the coordinate position of the border and its width and height at the same time.

### 3.4 Proposed Framework MindSpore

We implement our PLB method in PyTorch for research and exploration. At the same time, we recommend using an implementation version under the MindSpore framework as the final application. MindSpore is an enterprise-level application framework based on Huawei’s AI ecosystem. It has been used by Huawei in the medical field, and has open sourced the code of many detection models. This framework is an open-sourced product in the AI field that Huawei has been promoting in the past two years. Based on the hardware environment of Huawei’s Ascend series chips, it optimizes a large number of calculations in the model, speeds up the training and inference of the model. Due to the framework is easy to develop, efficient for execution and its full scene coverage, it can bring higher application value to our PLB method.

The implementation of our method in the MindSpore framework is basically the same as that under PyTorch, but we need to do some extra processing on the PLB weight computation. In order to prevent that all predict box areas may be zero during the training process, the area value of the predict box need to add by 1 to avoid division by zero exceptions.

## 4 Experiment Results Analysis

We use the dataset Pascal VOC2007 to explore the right way to apply our PLB methods, and use the BCCD blood cell detection dataset (Banik et al., 2020) to verify the effectiveness of PLB for medical image detection. Through exploration the effectiveness of our methods on Pascal VOC 2007, we verify it on BCCD datasets with our implementations both on PyTorch and MindSpore (https://www.MindSpore.cn/en). In our comparison experiment group, we use faster-rcnn model as the reference, chose resnet50 and FPN as the backbone network, and basically set the default values in the PyTorch library for its hyper-parameters, the SGD optimizer with momentum = 0.9, and the initial learning rate lr = 0.005, the adjustment step size of the learning rate step_size = 3.

The source code was released at: https://gitee.com/hubindijun/faster-rcnn-plb-MindSpore.git (MindSpore version); https://github.com/hubindijun/faster-rcnn-plb-PyTorch.git (PyTorch version).

### 4.1 Exploration of PLB in Natural Image Detection

The experiment uses the Pascal VOC2007 dataset (5011 images for training and 4,952 images for validation, 20 different categories). Then we evaluate our method with the coco evaluation standard. Finally, we mainly focus on the MAP and the detection accuracy of objects with different sizes to analyze the effect of PLB. The area range of small objects is (0,32*32), the area range of medium-sized objects is [32*32,96*96] and the area range of large objects is greater than 96*96, using pixel point number as the unit of object size. In the dataset, the ratio of small, medium and large objects is 845:2,698:4,301 in the training dataset, while the ratio in the testing dataset is 909:2,706:4,203.

We selectively perform PLB operations on different parts of the loss function. When the training epoch is 10, both the original model and new one reach a status of convergence.

PLB method in the four different loss components are named as follows, all of the four PLB operations use predict box as the default standard for size representation.

**PLB1C:** PLB in the first RPN stage of the detection model within coarse-grained classification loss;

**PLB1B:** PLB in the first RPN stage of the detection model within bounding box regression loss;

**PLB2C:** PLB in the second stage of the detection model within fine-grained classification loss;

**PLB2B:** PLB in the second stage of the detection model within further bounding box regression loss.

#### 4.1.1 The Selection of Predict Box or Matched Labeled Box for Size Representation

Firstly, we conduct the two experiments about PLB2C with default predict box and matched labeled box as the standard for size representation. The training accuracy effect are showed in Table 1. The results of the PLB2C shows that only use PLB operation in the fine-grained classification loss can significantly improve the detection accuracy of small objects, but the overall accuracy of the model is reduced. PLB2C means higher requirements for small objects and reduces the expectation of the detection effect of medium and large objects. Although the detection accuracy of small objects gets improved, the detection accuracy of medium and large ones will decrease. Finally, due to the proportion of small objects is relatively small in the dataset, the overall detection accuracy will also decrease in the training.

**TABLE 1:** Training accuracy effect of PLB2C (IOU = 0.50:0.95) on PyTorch.

	MAP	AP_small	AP_ Medium	AP_large
Original faster_rcnn	48.9	18.3	39.7	56.3
PLB2C (default predict box)	46.6 (−4.70%)	19.2 (+4.92%)	38.8 (−2.27%)	53.9 (−4.26%)
PLB2C (matched labeled box)	47.7 (−2.45%)	16.8 (−8.20%)	38.3 (−3.53%)	55.1 (−2.13%)

However, after replacing predict box with matched labeled box as the representation of the object size, the detection effect is reduced, even the detection effect for small objects is reduced by 8.2% as shows in Table 1. We can draw a conclusion that compare to the predict box, the matched labeled box is not suitable for the representation of the object size in the model training process.

#### 4.1.2 Ablation Experiments Analysis

Through our design and experiments, we summarize the detection effects of each scheme on the accuracy of small objects, as shown in Table 2. Comparing the results of each scheme, all of the PLB methods can obviously improve the detection accuracy of small objects. However, the detect effect of the PLB methods is different for medium and large objects. 

**TABLE 2:** Comparison of the PLB methods (IOU = 0.50:0.95) on PyTorch.

PLB1C	PLB1B	PLB2C	PLB2B	MAP	AP_small	AP_medium	AP_large
Not used	-	-	-	48.9	18.3	39.7	56.3
✓	-	-	-	49.3	19.3 (+5.46%)	40.2	56.5
-	✓	-	-	49.2	19.1 (+4.37%)	39.9	56.8
-	-	✓	-	46.6	19.2 (+4.92%)	38.8	53.9
-	-	-	✓	48.7	19.0 (+3.83%)	39.2	56.2
✓	-	✓		49.3	18.6 (+1.64%)	39.9	56.7
✓	✓	-	-	49.1	19.3 (+5.46%)	39.9	56.5

**FIGURE 2:**
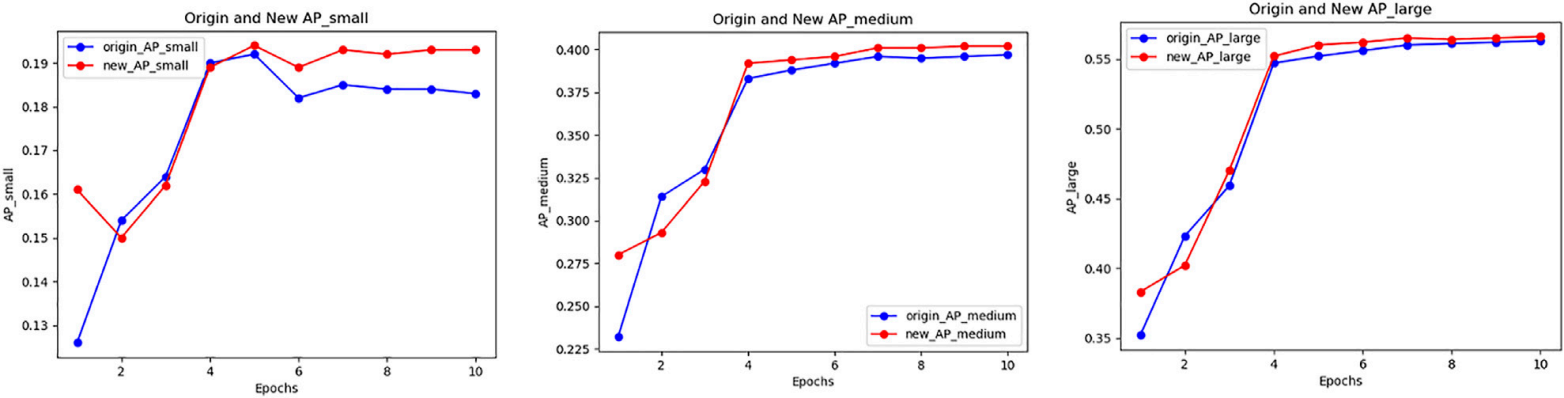
Accuracy variation of small, medium and large object in PLB1C.

With method PLB1C or PLB1B, the overall effect of the model keeps well, especially the detection accuracy of small objects has a significant improvement. Meanwhile, the methods have little impact on medium and large objects. The training accuracy tendency of PLB1C is shown in Figure 2.

**FIGURE 3:**
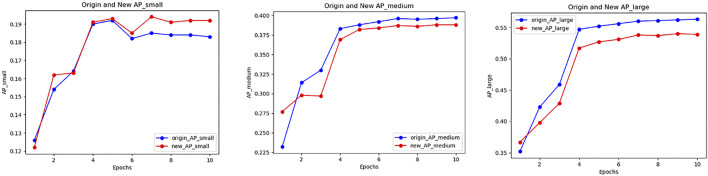
Accuracy variation of small, medium and large object in PLB2C.

 PLB operations in the second stage also improve the detection accuracy of small objects, as the results of PLB2C and PLB2B. However, that methods have negative impact on detection of medium and large objects. Due to there are more samples of medium and large objects than small ones, the overall detection accuracy is not well. As the accuracy tendency of PLB2C shows us in Figure 3.

Through the different results of the PLB methods, we can draw a conclusion that in the training process of the two-stage detector, the PLB operation utilized in the RPN stage can improve the detection effect of small objects and maintain the accuracy of medium and large objects. The purpose of PLB operation is to balance the contribution to the model loss of objects which have different sizes during the training process. In particular, we can adjust the design of the PLB factor so that the training of the model is transformed towards the detection accuracy improvement of small objects.

So how does PLB methods be more effective in the first stage? We guess that in the first stage of the training process, the coarse-grained classification task is mainly to classify the inspected objects as foreground or background, among them the background will not be included in the subsequent loss calculation. The smaller the object is, the easier it is to be misclassified as a background. So that in the second stage of detection, it is no longer involved in training process. Therefore, PLB method has a relatively obvious effect in the RPN stage of the two-stage detection model. Moreover, compared with the transformation of border regression loss with PLB, the loss transformation effect for the classification with PLB is more effective.

### 4.2 Practice of PLB in Medical Image Detection

**FIGURE 4:**
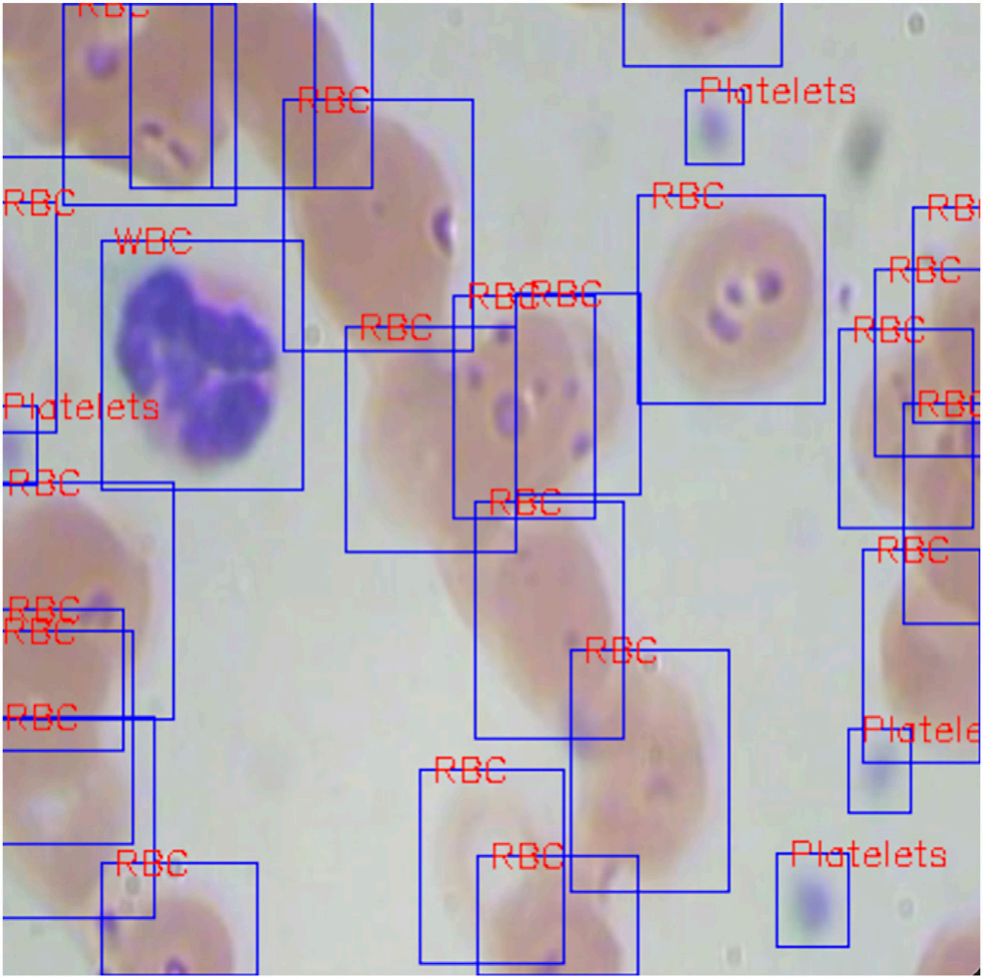
Blood cell detection example from the BCCD data set. The red tags denote different classifications of the detected objects.

We have verified that PLB has a certain adjustment effect for object detection, and using PLB in the RPN stage is more effective. Our design can also be used in specific medical application scenarios, such as routine blood testing and breast cancer diagnosis through lymphocyte detection. All of these application scenarios are based on detecting and measuring various types of blood cells to assist disease diagnosis. We use the BCCD data set (765 pictures for training, and 73 pictures for evaluating) to check the effect of PLB in cell detection. Figure 4 shows the blood cell detection in general, where there are three different cell types, in which the platelet size is relatively small and hard to detect.

We carried out three sets of experiments with this dataset. Using PLB with the model only in the coarse-grained classification loss, or only in the RPN border regression loss, or both of them at the same time, to demonstrate the effectiveness of it in medical application scenarios. When the training epochs is 15, both the old and new models reach to convergence. We conduct the experiments with our implementation both on PyTorch version and MindSpore version. Tables 3 and Table 4 shows the final results of pixel level balance respectively to these three experiments.

**TABLE 3:** Accuracy effect with PLB in BBCD Dataset (IOU = 0.50:0.95) on PyTorch.

	MAP	AP_small	AP_ Medium	AP_large
Origin model	63.1	47.8	64.9	50.2
PLB1C	63.6	49.7 (+3.97%)	65.2 (+0.46%)	49.6 (−1.20%)
PLB1B	63.1	48.6 (+1.67%)	64.0 (−1.40%)	50.6 (+0.80%)
PLB1C + PLB1B	63.7	49.0 (+2.51%)	66.6 (+2.62%)	50.2 (+0%)

**TABLE 4:** Accuracy effect with PLB in BBCD Dataset (IOU = 0.50:0.95) on MindSpore.

	MAP	AP_small	AP_ Medium	AP_large
Origin model	62.9	48.7	65.4	48.0
PLB1C	63.3	51.4 (+5.54%)	65.6 (+0.31%)	48.3 (+0.63%)
PLB1B	63.1	49.6 (+1.85%)	64.2 (−1.83%)	50.3 (+4.79%)
PLB1C + PLB1B	63.6	50.4 (+3.49%)	66.6 (+1.83%)	49.2 (+2.50%)

**FIGURE 5:**
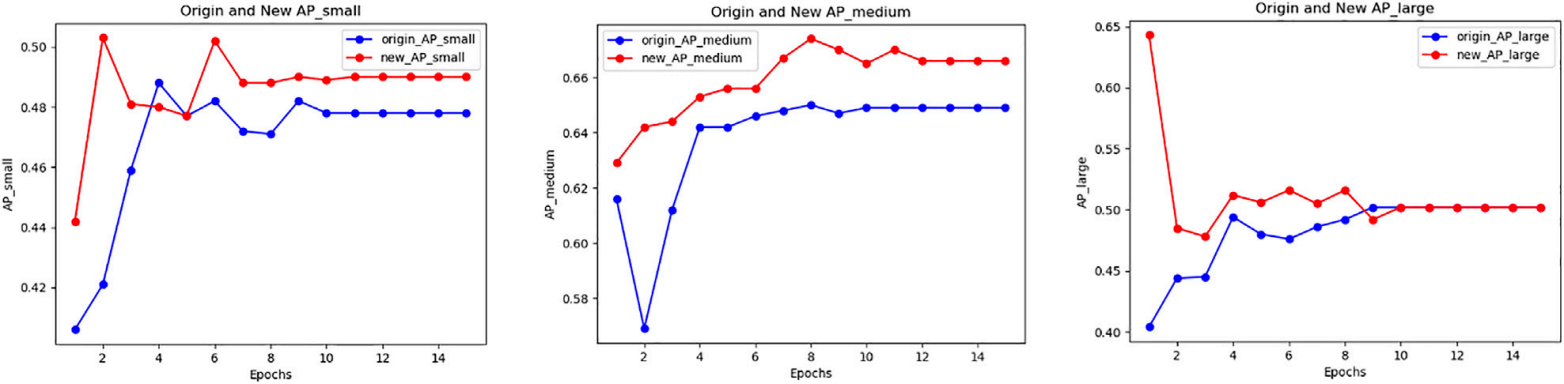
Accuracy variation of PLB1C combined with PLB1B within BCCD dataset.

Experimental results show that the PLB methods can effectively improve the detection effect of small objects in the process of medical image detection tasks. When using PLB in the two loss functions in the RPN stage at the same time, the overall detection effect is improved, especially for the detection accuracy of small objects. Figure 5 shows the accuracy variation of small, medium and large object when using PLB in the two loss components in the RPN stage.

## 5 Conclusions and Outlook

In this paper, we have proposed pixel level balance different from previous research, which focuses in the correlation of large and small objects in the training process. This method can be combined with other effective methods to improve small object detection, such as FPN network to improve the feature extraction, or data augmentation on the input dataset samples, etc. In some specific application scenarios, pixel level balance can provide more special effects. Obviously, in a train model, we can improve the detection accuracy of small objects while ignoring the large one by modifying the design of pixel level balance factors.

Pixel level balance can perform well in the problem of higher requirements for small objects in medical image detection. In our experiments, the effectiveness of this method for blood cell detection tasks has been demonstrated. It can be used in more other medical detection tasks in the future and achieve more development space or commercial value to medical image detection technology.

For future work, to alleviate the strong label requirement for deep learning-based detection, we would like to explore the possible way of applying visual matching-based approaches (Jiang et al., 2021a) for object detection and recognition. One promising technique is adopting graph matching with (higher-order) structure information (Yan et al., 2018) which can be more generalizable to new objects, and the detection may be performed in a joint matching fashion with multiple candidate objects with different techniques from heuristic optimization (Yan et al., 2015; Yan et al., 2016a) to dynamic programming based one (Jiang et al., 2021b). Moreover, the recently developed deep learning-based graph matching models (Wang et al., 2020; Wang et al., 2021) can also be explored which can better model the visual features for matching and object recognition. Readers are referred to the survey papers for more comprehensive study of these areas, in terms of both traditional learning-free methods (Yan et al., 2016b) as well as deep learning models (Yan et al., 2020). The hope is that a more structure information can be effectively used for object detection, against outliers, deformation, occlusion and other noise.

## Data Availability

Publicly available datasets were analyzed in this study. This data can be found here: Pascal VOC2007 http://host.robots.ox.ac.uk/pascal/VOC/voc2007/VOCtrainval_06-Nov-2007. tar BCCD https://public.roboflow.com/object-detection/bccd/4/download.
